# Supplementary material for: Bee Venom Melittin Disintegrates the Respiration of Mitochondria in Healthy Cells and Lymphoblasts, and Induces the Formation of Non-Bilayer Structures in Model Inner Mitochondrial Membranes
Source: Int J Mol Sci. 2021 Oct 15;22(20):11122. doi: 10.3390/ijms222011122 (PMC8538590; doi:10.3390/ijms222011122)
Supplement: Supplementary file 1 [file ijms-22-11122-s001.zip › ijms-1409410-supplementary.pdf]

Bee Venom Melittin Disintegrates Respiration of Mitochondria in Healthy and Leukemia Lymphocytes and Induces Formation of Non-bilayer Structures in Model Inner Mitochondrial Membranes

Edward Gasanoff, Yipeng Liu, Feng Li, Paul Hanlon, and Győző Garab

Supplementary materials

Table S1. Summary of the charged and polar groups of amino acid residues in the melittin binding sites that interact with polar head groups of truncated CL. Hypothetical binding sites in melittin that bind to the charged and polar groups of CL polar head were determined by AutoDock modeling. Designation of CL polar head groups is shown in Figure S1. Pb in $NH^{\delta+}_{pb}$ and $C{=}O^{\delta-}_{pb}$ denotes a peptide bond.

Binding site # and Affinity Energy Values	CL Polar Head Groups	Melittin a. a. Residues Interacting with CL Head	Bond Type
Binding site 1	a-PO_4^-	R22 ($CNH_2^{\delta+}$)	ion-polar
Affinity: –4.1 kcal/mol	b-PO_4^-	R22 ($CNH^{\delta+}C$)	ion-polar
	b-PO_4^-	Q26 ($CNH_2^{\delta+}$)	ion-polar
	$CO^{\delta-}H$	R22 ($CNH_2^{\delta+}$)	hydrogen
	$CO^{\delta-}H$	R22 ($CNH^{\delta+}C$)	hydrogen
	I-$C{=}O^{\delta-}$	R22($C{=}N^+H_2$)	ion-polar
	I-$C{=}O^{\delta-}$	S18($C–OH^{\delta+}$)	hydrogen
	IV-$C{=}O^{\delta-}$	Q25 ($CNH_2^{\delta+}$)	hydrogen
	4-I-$CO^{\delta-}C$	Q26 ($CNH_2^{\delta+}$)	hydrogen
Binding site 2	b-PO_4^-	R22($C{=}N^+H_2$)	ionic
Affinity: –4.0 kcal/mol	a-PO_4^-	R22 ($CNH_2^{\delta+}$)	ion-polar
	a-PO_4^-	R22 ($CNH^{\delta+}C$)	ion-polar
	b-PO_4^-	R22 ($CNH_2^{\delta+}$)	ion-polar
	$CO^{\delta-}H$	R22 ($CNH_2^{\delta+}$)	hydrogen
	I-$C{=}O^{\delta-}$	Q26 ($CNH_2^{\delta+}$)	hydrogen
Binding site 3	a-PO_4^-	W19 ($CNH^{\delta+}C$)	ion-polar
Affinity: –4.0 kcal/mol	b-PO_4^-	W19 ($CNH^{\delta+}C$)	ion-polar
	b-PO_4^-	Q26 ($CNH_2^{\delta+}$)	ion-polar
	b-PO_4^-	R22 ($CNH_2^{\delta+}$)	ion-polar
	b-PO_4^-	R22 ($CNH^{\delta+}C$)	ion-polar
	2-$CO^{\delta-}C$	R22 ($CNH_2^{\delta+}$)	hydrogen
	II-$C{=}O^{\delta-}$	R22($C{=}N^+H_2$)	ion-polar
	4-I-$CO^{\delta-}C$	Q26 ($CNH_2^{\delta+}$)	hydrogen
Binding site 4	II-$C{=}O^{\delta-}$	T11 ($C–OH^{\delta+}$)	hydrogen
Affinity:	IV-$C{=}O^{\delta-}$	W19 ($CNH^{\delta+}C$)	hydrogen

–3.9 kcal/mol	CL head hydro-<br>phobic surface	12G,13L,14P,<br>15A,16L,17I	hydrophobic
Binding site 5<br>Affinity: –3.9 kcal/mol	a-PO_4^-<br>b-PO_4^-<br>b-PO_4^-<br>I-$C{=}O^{\delta-}$<br>II-$C{=}O^{\delta-}$<br>$CO^{\delta-}H$<br>$CO^{\delta-}H$<br>4-$CO^{\delta-}C$	W19 ($CNH^{\delta+}C$)<br>R22 ($CNH^{\delta+}C$)<br>Q26 ($CNH_2^{\delta+}$)<br>R22 ($CNH_2^{\delta+}$)<br>R22 ($CNH_2^{\delta+}$)<br>R22 ($CNH_2^{\delta+}$)<br>R22 ($CNH^{\delta+}C$)<br>Q25 ($CNH_2^{\delta+}$)	ion-polar<br>ion-polar<br>ion-polar<br>hydrogen<br>hydrogen<br>hydrogen<br>hydrogen<br>hydrogen
Binding site 6<br>Affinity: –3.9 kcal/mol	a-PO_4^-<br>a-PO_4^-<br>a-PO_4^-<br>a-PO_4^-<br>1-$CO^{\delta-}C$<br>$CO^{\delta-}H$<br>b-PO_4^-	Q26 ($CNH_2^{\delta+}$)<br>R22 ($CNH_2^{\delta+}$)<br>R22 ($CNH^{\delta+}C$)<br>W19 ($CNH^{\delta+}C$)<br>W19 ($CNH^{\delta+}C$)<br>R22 ($CNH_2^{\delta+}$)<br>R22 ($CNH_2^{\delta+}$)	ion-polar<br>ion-polar<br>ion-polar<br>ion-polar<br>hydrogen<br>hydrogen<br>ion-polar
Binding site 7<br>Affinity: –3.9 kcal/mol	a-PO_4^-<br>a-PO_4^-<br>II-$C{=}O^{\delta-}$<br>$COH^{\delta+}$<br>b-PO_4^-	R24 ($C{=}N^+H_2$)<br>R24 ($CNH_2^{\delta+}$)<br>R24 ($CNH_2^{\delta+}$)<br>R24 ($C{=}O^{\delta-}{}_{pb}$)<br>R24 ($C{=}N^+H_2$)	ionic<br>ion-polar<br>hydrogen<br>hydrogen<br>ionic
Binding site 8<br>Affinity: –3.9 kcal/mol	b-PO_4^-<br>b-PO_4^-<br>b-PO_4^-<br>3-$CO^{\delta-}C$<br>$CO^{\delta-}H$<br>II-$C{=}O^{\delta-}$<br>II-$C{=}O^{\delta-}$	R22 ($C{=}N^+H_2$)<br>R22 ($CNH_2^{\delta+}$)<br>Q26 ($CNH_2^{\delta+}$)<br>Q26 ($CNH_2^{\delta+}$)<br>W19 ($CNH^{\delta+}C$)<br>R22 ($C{=}N^+H_2$)<br>R22 ($CNH_2^{\delta+}$)	ionic<br>ion-polar<br>ion-polar<br>hydrogen<br>hydrogen<br>ion-polar<br>hydrogen
Binding site 9<br>Affinity: –3.9 kcal/mol	a-PO_4^-<br>a-PO_4^-<br>b-PO_4^-<br>b-PO_4^-<br>3-$CO^{\delta-}C$<br>3-$CO^{\delta-}C$<br>III-$C{=}O^{\delta-}$<br>IV-$C{=}O^{\delta-}$	R22 ($C{=}N^+H_2$)<br>R22 ($CNH_2^{\delta+}$)<br>R22 ($CNH_2^{\delta+}$)<br>R22 ($CNH^{\delta+}C$)<br>R22 ($CNH^{\delta+}C$)<br>Q26 ($CNH_2^{\delta+}$)<br>Q26 ($CNH_2^{\delta+}$)<br>Q26 ($CNH_2^{\delta+}$)	ionic<br>ion-polar<br>ion-polar<br>ion-polar<br>hydrogen<br>hydrogen<br>hydrogen<br>hydrogen

Table S2. Summary of the charged and polar groups of amino acid residues in the melittin binding sites that interact with polar head groups of truncated PS. Hypothetical binding sites in melittin that bind to the charged and polar groups of PS polar head were determined by AutoDock modeling. Designation of PS polar head groups is shown in Figure S1. Pb in $NH^{\delta+}{}_{pb}$ denotes a peptide bond.

Binding Site # and Affinity Energy Values	PS Polar Head Groups	Melittin a. a. Residues Interacting with PS Head	Bond Type
Binding site 1<br>Affinity: –3.8 kcal/mol	PO_4^-	R22 ($CNH_2^{\delta+}$)	ion-polar
	I-C=$O^{\delta-}$	R22 (C=N^+H_2)	ionic
	I-C=$O^{\delta-}$	R22 ($CNH_2^{\delta+}$)	ion-polar
	II-C=$O^{\delta-}$	A15 ($NH^{\delta+}{}_{pb}$)	ion-polar
	COO^-	R22 ($CNH^{\delta+}C$)	ion-polar
	COO^-	Q26 ($CNH_2^{\delta+}$)	ion-polar
Binding site 2<br>Affinity: –3.7 kcal/mol	PO_4^-	R22 ($CNH^{\delta+}C$)	ion-polar
	PO_4^-	Q26 ($CNH_2^{\delta+}$)	ion-polar
	COO^-	R22 ($CNH_2{}^{\delta+}$)	ion-polar
	COO^-	R22 ($CNH^{\delta+}C$)	ion-polar
	COO^-	Q26 ($CNH_2^{\delta+}$)	ion-polar
	I-C=$O^{\delta-}$	Q25 ($CNH_2^{\delta+}$)	hydrogen
Binding site 3<br>Affinity: –3.6 kcal/mol	PO_4^-	L16 ($NH^{\delta+}{}_{pb}$)	ion-polar
	COO^-	R22 ($CNH_2{}^{\delta+}$)	ion-polar
	COO^-	W19 ($NH^{\delta+}{}_{pb}$)	ion-polar
	II-C=$O^{\delta-}$	L13 ($NH^{\delta+}{}_{pb}$)	hydrogen
Binding site 4<br>Affinity: –3.6 kcal/mol	COO^-	R22 (C=N^+H_2)	ionic
	COO^-	R22 ($CNH_2{}^{\delta+}$)	ion-polar
	I-C=$O^{\delta-}$	L13 ($NH^{\delta+}{}_{pb}$)	hydrogen
Binding site 5<br>Affinity: –3.6 kcal/mol	PO_4^-	Q25 ($CNH_2^{\delta+}$)	ion-polar
	COO^-	R22 (C=N^+H_2)	ionic
	II-C=$O^{\delta-}$	Q26 ($CNH_2^{\delta+}$)	hydrogen
	2-$CO^{\delta-}C$	Q26 ($CNH_2^{\delta+}$)	hydrogen
Binding site 6<br>Affinity: –3.6 kcal/mol	PO_4^-	K21 (CN^+H_3)	ionic
	I-C=$O^{\delta-}$	K21 (CN^+H_3)	ion-polar
	N^+H_3	S18 ($CO^{\delta-}H$)	ion-polar
	COO^-	Q25 ($CNH_2^{\delta+}$)	ion-polar
Binding site 7<br>Affinity: –3.6 kcal/mol	PO_4^-	R22 (C=N^+H_2)	ionic
	PO_4^-	S18 ($COH^{\delta+}$)	ion-polar
	1-$CO^{\delta-}C$	R22 (C=N^+H_2)	ion-polar
	1-$CO^{\delta-}C$	S18 ($COH^{\delta+}$)	hydrogen
	I-C=$O^{\delta-}$	R22 ($CNH_2{}^{\delta+}$)	hydrogen

	2-CO$^{\delta-}$C	S18 (COH$^{\delta+}$)	hydrogen
Binding site 8<br>Affinity: –3.5 kcal/mol	COO$^-$<br>N$^+$H$_3$	K7 (CN$^+$H$_3$)<br>T11 (CO$^{\delta-}$H)	ionic<br>ion-polar
Binding site 9<br>Affinity: –3.5 kcal/mol	COO$^-$<br>II-C=O$^{\delta-}$<br>II-C=O$^{\delta-}$	L13 (NH$^{\delta+}_{pb}$)<br>R22 (CNH$_2$ $^{\delta+}$)<br>R22 (C=N$^+$H$_2$)	ion-polar<br>hydrogen<br>ion-polar

Table S3. Summary of the charged and polar groups of amino acid residues in the melittin binding sites that interact with polar head groups of truncated PC. Hypothetical binding sites in melittin that bind to the charged and polar groups of PC polar head were determined by AutoDock modeling. Designation of PC polar head groups is shown in Figure S1. Pb in NH$^{\delta+}_{pb}$ denotes a peptide bond.

**Binding Site # and Affinity Energy Values**	**PC Polar Head Groups**	**Melittin a. a. Residues Interacting with PC Head**	**Bond Type**
Binding site 1<br>Affinity: –3.3 kcal/mol	PO$_4^-$<br>PO$_4^-$<br>PO$_4^-$<br>PO$_4^-$<br>1-CO$^{\delta-}$C<br>2-CO$^{\delta-}$C	W19 (CNH$^{\delta+}$C)<br>R22(CNH$_2^{\delta+}$)<br>R22 (CNH$^{\delta+}$C)<br>Q26 (CNH$_2^{\delta+}$)<br>Q26 (CNH$_2^{\delta+}$)<br>Q26 (CNH$_2^{\delta+}$)	ion-polar<br>ion-polar<br>ion-polar<br>ion-polar<br>hydrogen<br>hydrogen
Binding site 2<br>Affinity: –3.2 kcal/mol	PO$_4^-$<br>PO$_4^-$<br>PO$_4^-$	R22 (C=N$^+$H$_2$)<br>R22(CNH$_2^{\delta+}$)<br>S18 (COH$^{\delta+}$)	ionic<br>ion-polar<br>ion-polar
Binding site 3<br>Affinity: –3.2 kcal/mol	II-C=O$^{\delta-}$<br>PC head hydro-phobic surface	W19 (CNH$^{\delta+}$C)<br>V8, L13, A14, L16	hydrogen<br>hydrophobic
Binding site 4<br>Affinity: –3.2 kcal/mol	II-C=O$^{\delta-}$<br>PO$_4^-$<br>PC head hydro-phobic surface	G12 (NH$^{\delta+}_{pb}$)<br>L16 (NH$^{\delta+}_{pb}$)<br>V8, W19	hydrogen<br>ion-polar<br>hydrophobic
Binding site 5<br>Affinity: –3.1 kcal/mol	PO$_4^-$<br>PO$_4^-$<br>II-C=O$^{\delta-}$	R22(CN$^+$H$_2$)<br>R22(CNH$_2^{\delta+}$)<br>W19 (CNH$^{\delta+}$C)	ionic<br>ion-polar<br>hydrogen
Binding site 6<br>Affinity: –3.1 kcal/mol	II-C=O$^{\delta-}$<br>PC head hydro-phobic surface	K21 (N$^+$H$_3$)<br>L6, L9, L13	ion-polar<br>hydrophobic
Binding site 7	I-C=O$^{\delta-}$	R22(C=N$^+$H$_2$)	ion-polar

Affinity: –3.1 kcal/mol	II-C=O$^{\delta-}$<br>PC head hydro-phobic surface	W19 (CNH$^{\delta+}$C)<br>V8, L13, L16	hydrogen<br>hydrophobic
Binding site 8<br>Affinity: –3.1 kcal/mol	I-C=O$^{\delta-}$<br>II-C=O$^{\delta-}$<br>II-C=O$^{\delta-}$<br>PC head hydro-phobic surface	W19 (CNH$^{\delta+}$C)<br>R22(C=N^{+}H$_2$)<br>R22(CNH$_2^{\delta+}$)<br>V8, L13, L16	hydrogen<br>ion-polar<br>hydrogen<br>hydrophobic
Binding site 9<br>Affinity: –3.1 kcal/mol	PO$_4^-$<br>PO$_4^-$<br>PO$_4^-$<br>1-CO$^{\delta-}$C<br>2-CO$^{\delta-}$C	R22(CNH$_2^{\delta+}$)<br>R22 (CNH$^{\delta+}$C)<br>Q26 (CNH$_2^{\delta+}$)<br>Q26 (CNH$_2^{\delta+}$)<br>Q26 (CNH$_2^{\delta+}$)	ion-polar<br>ion-polar<br>ion-polar<br>hydrogen<br>hydrogen

Table S4. Summary of the charged and polar groups of amino acid residues in the melittin binding sites that interact with polar head groups of truncated PA. Hypothetical binding sites in melittin that bind to the charged and polar groups of PA polar head were determined by AutoDock modeling. Designation of PA polar head groups is shown in Figure S1. Pb in NH$^{\delta+}_{pb}$ denotes a peptide bond.

**Binding Site # and Affinity Energy Values**	**PA Polar Head Groups**	**Melittin a. a. Residues Interacting with PA Head**	**Bond Type**
Binding site 1<br>Affinity: –3.5 kcal/mol	PO$_4^-$<br>PO$_4^-$<br>I-C=O$^{\delta-}$	R22 (C=N^{+}H$_2$)<br>R22 (CNH$_2^{\delta+}$)<br>W19 (CNH$^{\delta+}$C)	ionic<br>ion-polar<br>hydrogen
Binding site 2<br>Affinity: –3.4 kcal/mol	PO$_4^-$<br>PO$_4^-$	R22 (C=N^{+}H$_2$)<br>R22 (CNH$_2^{\delta+}$)	ionic<br>ion-polar
Binding site 3<br>Affinity: –3.4 kcal/mol	PO$_4^-$<br>II-C=O$^{\delta-}$<br>II-C=O$^{\delta-}$	L16 (NH$^{\delta+}_{pb}$)<br>R22 (C=N^{+}H$_2$)<br>R22 (CNH$_2^{\delta+}$)	ion-polar<br>ion-polar<br>hydrogen
Binding site 4<br>Affinity: –3.3 kcal/mol	PO$_4^-$<br>PO$_4^-$<br>1-CO$^{\delta-}$C	R22 (C=N^{+}H$_2$)<br>R22 (CNH$_2^{\delta+}$)<br>R22 (CNH$_2^{\delta+}$)	ionic<br>ion-polar<br>hydrogen
Binding site 5<br>Affinity: –3.3 kcal/mol	PO$_4^-$<br>PO$_4^-$<br>PO$_4^-$<br>1-CO$^{\delta-}$C<br>2-CO$^{\delta-}$C	R22 (CNH$_2^{\delta+}$)<br>R22 (CNH$^{\delta+}$C)<br>Q26 (CNH$_2^{\delta+}$)<br>Q26 (CNH$_2^{\delta+}$)<br>Q26 (CNH$_2^{\delta+}$)	ion-polar<br>ion-polar<br>ion-polar<br>hydrogen<br>hydrogen

Binding site 6<br>Affinity: –3.3 kcal/mol	PO_4^-<br>PO_4^-<br>PO_4^-	R22 ($C{=}N^+H_2$)<br>R22 ($CNH_2^{\delta+}$)<br>S18 ($COH^{\delta+}$)	ionic<br>ion-polar<br>ion-polar
Binding site 7<br>Affinity: –3.2 kcal/mol	PO_4^-<br>1-$CO^{\delta-}C$	R22 ($CNH_2^{\delta+}$)<br>W19 ($CNH^{\delta+}C$)	ion-polar<br>hydrogen
Binding site 8<br>Affinity: –3.2 kcal/mol	PO_4^-<br>I-$C{=}O^{\delta-}$<br>II-$C{=}O^{\delta-}$	K21 (CN^+H_3)<br>Q25 ($CNH_2^{\delta+}$)<br>S18 ($COH^{\delta+}$)	ion-polar<br>hydrogen<br>hydrogen
Binding site 9<br>Affinity: –3.2 kcal/mol	I-$C{=}O^{\delta-}$<br>II-$C{=}O^{\delta-}$<br>PO_4^-	R22 ($CNH_2^{\delta+}$)<br>W19 ($CNH^{\delta+}C$)<br>L16 ($NH^{\delta+}{}_{pb}$)	hydrogen<br>hydrogen<br>ion-polar

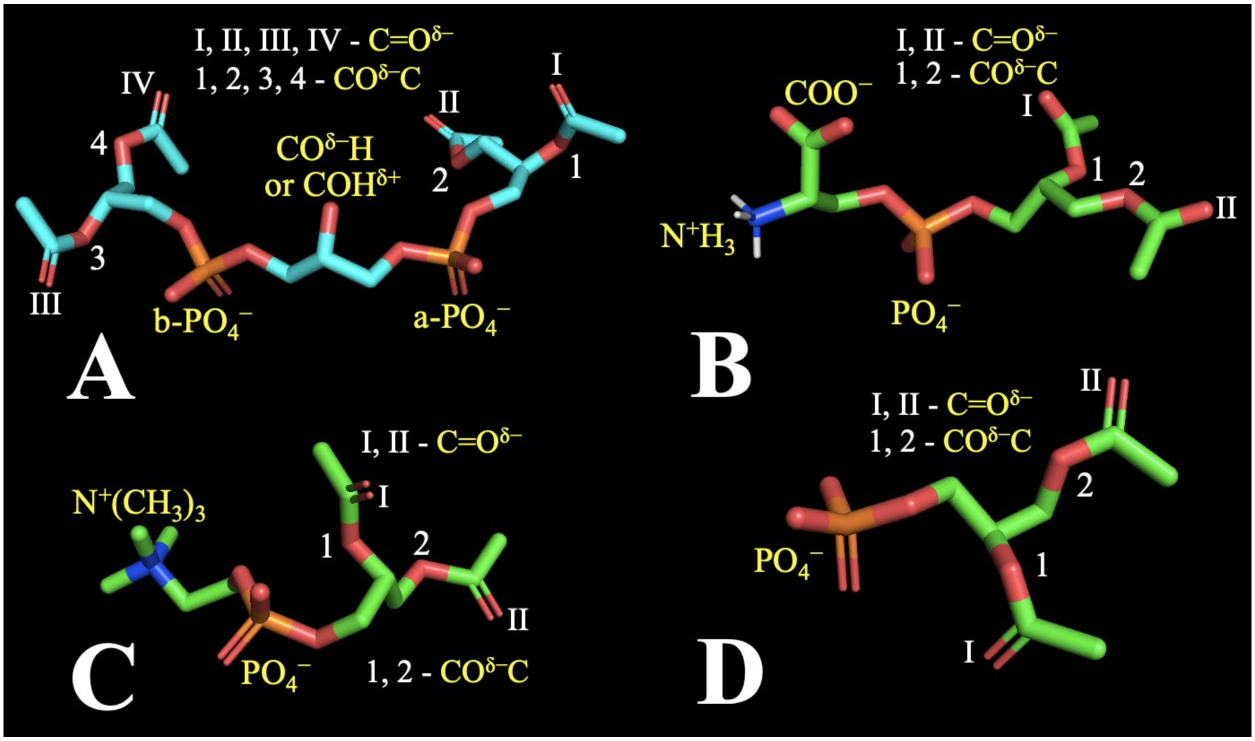

Figure S1. Designation of polar head groups (charged and dipolar) of cardiolipin (**A**), phosphatidylserine (**B**), phosphatidylcholine (**C**), and phosphatidic acid (**D**).
